# Thermoelectric Properties of NbCoNi_x_Sn (x = 0–1)

**DOI:** 10.3390/ma18133189

**Published:** 2025-07-05

**Authors:** Moritz Thiem, Ruijuan Yan, Anke Weidenkaff, Wenjie Xie

**Affiliations:** Materials and Resources, Department of Materials Science, Technical University of Darmstadt, 64287 Darmstadt, Germany; moritz.thiem@mr.tu-darmstadt.de (M.T.); kate66998@gmail.com (R.Y.); anke.weidenkaff@mr.tu-darmstadt.de (A.W.)

**Keywords:** thermoelectric materials, half-Heusler, NbCoSn, microstructural modification, secondary phase

## Abstract

The half-Heusler (HH) compound NbCoSn, with 18 valence electrons, is a promising thermoelectric (TE) material due to its favourable electrical properties and excellent thermal and chemical stability. Enhancing its TE performance typically involves doping and microstructure engineering. In this study, Ni was introduced into NbCoSn to form NbCoNi_x_Sn (x = 0–1), and the effects of Ni content on the microstructure and TE properties were systematically investigated. At low doping levels (x ≤ 0.05), Ni occupies interstitial sites, forming NbCoNi_x_Sn solid solutions. At higher concentrations (x > 0.05), full-Heusler (FH) secondary phases emerge, resulting in HH–FH composites. The introduction of Co/Ni interstitials enhances TE performance by creating in-gap electronic states and increasing phonon scattering through point defects. A clear structural transition from HH to FH phases is observed with increasing Ni content. The highest figure of merit, *ZT* ≈ 0.52 at 975 K, was obtained for NbCoNi_0.05_Sn, comparable to the best values reported for this system.

## 1. Introduction

In our modern world, global climate change is the essential driver for research on sustainable energy. Global energy consumption growth leads to the need for new low-carbon energy sources [1,2]. Thermoelectric materials can directly convert waste heat into electricity and offer a way to improve the sustainability of already existing systems, such as combustion engines, sunlight, or by-products of other heat-producing processes [3,4,5,6]. Moreover, thermoelectrics offer various flexible applications such as temperature control, refrigeration, and power generation [7,8,9]. The dimensionless thermoelectric figure of merit *ZT* = *σS*^2^/(*κ*_L_ + *κ*_e_) (with *σ* being the electrical conductivity, *S* the Seebeck coefficient, and *κ*_e_ and *κ*_L_ the contributions of electrons and lattice to the total thermal conductivity *κ*_tot_) is used to determine the effectiveness of the system.

The class of half-Heusler (HH) compounds have demonstrated promising thermoelectric performance, with peak figures of merit (*ZT*) reaching values as high as 1.5 [10,11,12,13]. A key advantage of these materials lies in their tuneable electronic properties, which can be adjusted through compositional modifications without compromising the integrity of their crystal structure [14,15]. Moreover, HH compounds are also attractive for practical applications due to their low toxicity, high mechanical robustness, and excellent thermal stability [16,17,18,19]. As a result, current research efforts are focused on further enhancing the thermoelectric efficiency of HH materials. This can be achieved either by increasing the power factor (*PF = S^2^σ*) or by reducing their inherently high thermal conductivity *κ* [19,20]. To this end, various approaches have been explored, including nanostructuring [17], the introduction of point defects [21], plastic deformation [22], and band structure engineering [23].

NbCoSn is a member of the half-Heusler (HH) compound family and has shown promising thermoelectric (TE) performance, with reported *ZT* values in the range of 0.5 to 0.6 [24,25,26]. It is a ternary half-Heusler compound that crystallises in the cubic MgAgAs-type structure (space group *F*$\bar{43}$*m*, No. 216) [27,28]. NbCoSn is an intrinsic *n*-type semiconductor with an 18 valence electron count (VEC) and a bandgap of approximately 0.987 eV. This bandgap corresponds to roughly 10 *k_B_T*_0_, where *k_B_* is the Boltzmann constant and *T_0_* is the application temperature, making NbCoSn a promising candidate for high-temperature thermoelectric applications [29]. So far, many studies have been carried out to improve the TE properties of NbCoSn compounds via tuning the chemical compositions, such as Sb substitution on the Sn site [24], Pt substitution on the Co site [25], and Ta substitution on the Nb site [26].

**Figure 1:**
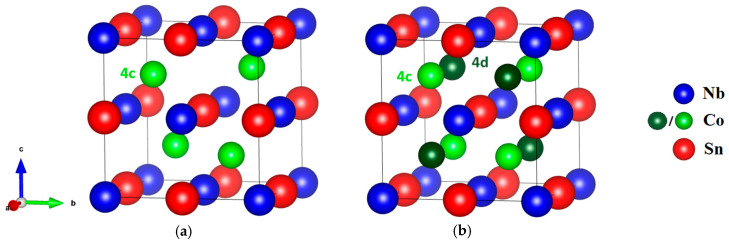
Crystal structure of (**a**) HH NbCoSn compound and (**b**) FH NbCo_2_Sn compound.

Among all the studies, it was noticed that excess Co is always observed in directly melted NbCoSn samples, synthesised by high-temperature techniques (arc-melting or induction-melting), despite being designed as the nominal Nb:Co:Sn = 1:1:1 composition [16,25,30,31,32], indicating that there are some stable intrinsic interstitial Co defects. This excess in Co content originates from the formation of secondary phases, mainly Nb_3_Sn and Sn, during the melting procedure and cannot be fully eliminated. It has been reported that interstitial Co defects can introduce in-gap states between the conduction and valence bands, thereby significantly enhancing electrical conductivity [33,34]. Recently, Yan et al. [16] demonstrated that in NbCo_1−x_Ni_x_Sn compounds, Ni atoms not only substituted on the 4c lattice position of Co but also occupied the vacant 4d position in the half-Heusler lattice, leading to the partial formation of the full-Heusler (FH) phase in the half-Heusler matrix (Figure 1). The occupation of the 4d site with Ni was verified by theoretical calculations, which also showed that interstitial Ni defects can create in-gap states that can improve the thermoelectric performance. Although these point defects can enhance phonon scattering, the reported thermal conductivities were still higher than comparable literature values [25], limiting the overall improvement in *ZT*.

To further enhance the *ZT* and understand the role of Ni in the system, NbCoNi_x_Sn (*x* = 0–1) samples are prepared by arc-melting, followed by additional ball milling and spark plasma sintering. Laboratory X-ray Diffraction (XRD) and Scanning Electron Microscopy (SEM) were employed to show the change in sample composition from a pure NbCoSn HH phase to a NbCoNiSn FH phase with negligible secondary phases. Due to the introduction of Ni, electrical properties are enhanced at low doping levels, leading to a maximum *ZT* of ~0.52 at 975 K. Moreover, the introduction of ball milling to the NbCoNi_x_Sn systems proves to be a valuable addition to the synthesis since thermal conductivities were reduced drastically.

## 2. Experimental Methods

NbCoNi_x_Sn (with nominal compositions x = 0, 0.1, 0.05, 0.1, 0.15, 0.2, 0.25, 0.3, 0.4, 0.5, and 1.0) synthesis starts by arc-melting the stoichiometric amounts of Nb (wire, 99.999%), Co (bulk, 99.999%), Ni (wire, 99.99%), and Sn (shot, 99.99%) under an inert argon atmosphere. To ensure a homogeneous melting process, the ingots are flipped, crushed, and re-melted several times. Afterwards, the bulk samples were ground into powder using high-energy ball milling (Pulverisette 7, Fritsch, Idar-Oberstein, Germany) for 2 h. The powders were loaded in a Ø20 mm graphite die and spark plasma sintered at 1223 K for 5 min. Subsequently, the cylinder-shaped samples are cut into discs and bars for phase characterisation, structural analysis, and TE property measurements.

The powder X-ray diffraction (XRD) patterns were all measured using Mo Kα1 radiation in a STOE STAD diffractometer (Darmstadt, Germany). For analysis of phase composition and phase distribution, Scanning Electron Microscopy is used (SEM, TESCAN, VEGA 3, Dortmund, Germany) with an energy-dispersive X-ray spectrometer (EDX, EDAX Genesesis, AMTEK Gmbh, Unterschleissheim, Germany). For high-temperature measurements of the Seebeck coefficient *S* and electrical resistivity σ, a ZEM-3 device (ULVAC-RIKO, Yokohama, Japan) was used. The charge-carrier concentration *n*_H_ and mobility *µ*_H_ were measured at room temperature with an applied magnetic field of −520 to 520 mT by a commercial Hall measurement system HT-Hall (Fraunhofer IPM, Freiburg, Germany). Measurements of the thermal conductivity were performed based on the formula *κ = DdC_p_*, with *D* being the thermal diffusivity, *d* being the densities of the samples, and *C_p_* being the specific heat. *D* and *d* were, respectively, measured by laser flash analysis (LFA 457, NETZSCH, Selb, Germany) and an Archimedes kit. *C_p_* was determined using the Dulong–Petit law. Uncertainties for characterisation techniques for thermoelectric properties are ±3% for *σ*, ±5% for *S*, and ±10% for *κ*.

## 3. Results and Discussion

### 3.1. Phase Composition and Microstructure Characterisation

**Figure 2:**
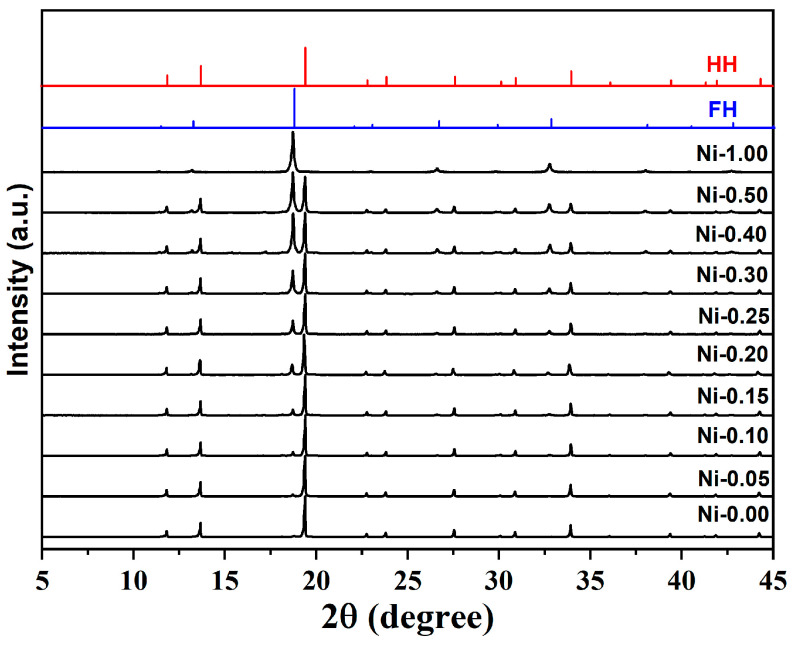
Powder XRD pattern of NbCoNi_x_Sn patterns after SPS with literature peaks of half-Heusler (HH) and full-Heusler (FH) phases.

The laboratory XRD patterns of nominal NbCoNi*_x_*Sn samples (x = 0, 0.05, 0.1, 0.15, 0.2, 0.25, 0.3, 0.4, 0.5, and 1; hereafter referred to as Ni-*x* for simplicity) are shown in Figure 2. The main phases are consistent with the MgAgAs-type HH structure and the AlCu_2_Mn-type FH structure. Typical secondary phases for these compounds are Nb_3_Sn, Co_7_Nb_6_, and elemental Sn [24,35]. Hardly any secondary-phase patterns were observed in the XRD shown in Figure 2, with intensities too low to provide precise information on their phase identity and quantity. The intensities of secondary phases (Nb_3_Sn, Co_7_Nb_6_, and Sn) do not enhance with Ni contents, indicating the secondary phases in all Ni-*x* samples are more or less the same. The main change in XRD patterns is the increase in intensity of the FH-phase peaks with increasing Ni content. Previous studies on NbCo_1-x_Ni_x_Sn systems demonstrated that Ni occupying the vacant 4d position is energetically favourable. Furthermore, the introduction of Ni can stabilise the FH phase (NbCo_2-x_Ni_x_Sn), and it is more stable than the NbCo_2_Sn phase [16]. Therefore, the increase in the FH phase in the NbCoNi_x_Sn system was expected as the quantity of the FH phase is directly related to the amount of excess Ni introduced into the system.

The linear increase in the FH phase with increasing Ni content is also reflected in the measured densities (Table 1). Overall, the measured densities were close to the theoretical densities. For the theoretical density calculations, it was assumed that the nominal excess Ni occupies the 4d lattice site. The good agreement between measured and theoretical densities supports the validity of this assumption.

**Figure 3:**
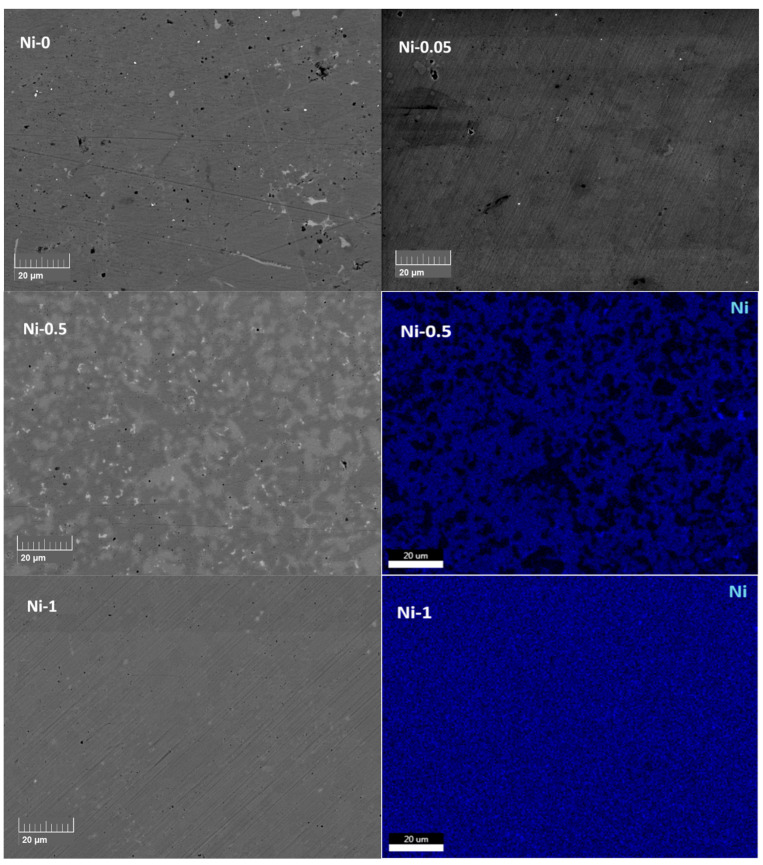
BSE image and corresponding EDX elemental mapping of Ni of representative NbCoNi_x_Sn compounds.

To further investigate phase distribution and microstructure in the Ni-*x* compounds, back-scattered electron (BSE) imaging and corresponding energy-dispersive X-ray (EDX) mapping of Ni were conducted on polished sample surfaces, and typical results are presented in Figure 3. The EDX analysis results for the two main phases are summarised in Table 2. An excess of Co was detected in nearly all phases. This excess can be attributed to the formation of secondary phases such as Nb_3_Sn and elemental Sn, which result from the high-temperature melting synthesis. These secondary phases are difficult to eliminate using conventional melting methods and contribute to an excess of Co in the remaining Heusler phases, even when the nominal composition is adjusted accordingly [16,25,30,31,32]. Regarding the effect of adding Ni into the NbCoSn system, the volume fraction of the FH phase increases with increasing Ni content and is homogeneously distributed within the HH matrix. EDX Ni mapping confirms that the FH phase is the primary host for Ni, while point analyses reveal only trace amounts of Ni in the HH phase. Fracture surface SEM imaging was also carried out to estimate grain sizes (Supporting Information Figure S1), which were found to be below 10 µm for all samples. This is approximately an order of magnitude smaller than those reported in earlier studies on NbCoNiSn compounds [16].

### 3.2. Transport Properties

**Figure 4:**
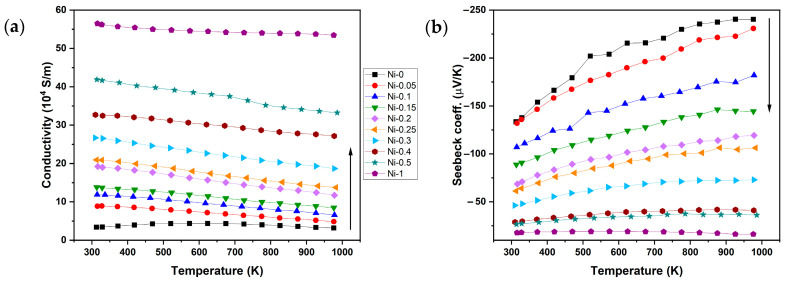
Temperature dependence measurements of (**a**) electrical conductivity and (**b**) Seebeck coefficient.

**Figure 5:**
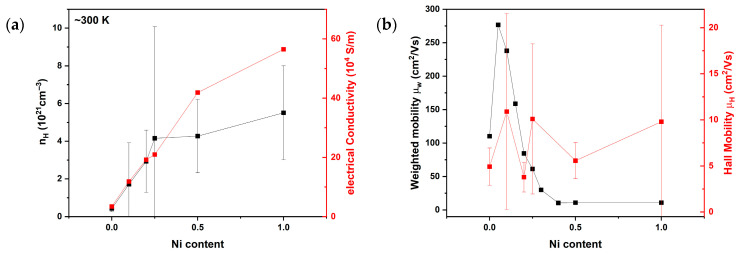
Dependences of (**a**) the charge-carrier concentration and electrical conductivity, and (**b**) weighted mobility and Hall mobility of the Ni content in NbCoNi_x_Sn compound at room temperature.

The results of the thermoelectric property measurements are displayed in Figure 4. For all samples, electrical conductivity decreases with increasing temperature, indicating a metal-like transport behaviour. At room temperature, the electrical conductivity increases almost linearly with Ni content from 4.69∙10^4^ S/m for Ni-0 to 53.41∙10^4^ S/m for Ni-1. Conversely, the Seebeck coefficient decreases with increasing Ni content. This inverse relationship between the electrical conductivity and Seebeck coefficient suggests an increase in charge-carrier concentration with higher Ni content, consistent with the expected interdependence of these properties. To verify this, Hall effect measurements were performed, and the results are shown in Figure 5. Hall measurements (Figure 5) confirm the assumption of an increase in charge-carrier concentration with higher Ni content. The observed trends in the electrical conductivity and Seebeck coefficient can be attributed to the evolving phase composition, specifically the increasing proportion of the full-Heusler phase relative to the half-Heusler phase. The FH phase has an intrinsically low Seebeck coefficient (sample Ni-1, Figure 4b) but a higher electrical conductivity, and vice versa for the HH phase. This interpretation is consistent with the microstructural analysis from SEM, which revealed a progressive increase in FH phase content with increasing Ni addition.

The observed increase in charge-carrier concentration in this system can arise through several mechanisms. First, the substitution of Co with Ni introduces an additional electron per Ni atom. Second, excess Ni can occupy interstitial 4d sites, forming in-gap states that reduce the band gap and thereby increase carrier concentration—a phenomenon previously confirmed in other HH systems such as MNiSn (M = Ti, Zr, Hf) [36,37]. Third, the formation of the half-metallic full-Heusler (FH) phase also contributes free carriers to the system [38].

Among these, direct substitution of Co by Ni is considered energetically less favourable than interstitial occupation of the 4d site [16], and can therefore be excluded as a dominant mechanism. Given the continued increase in electrical conductivity and decrease in Seebeck coefficient with increasing FH phase content, the FH phase is identified as the primary contributor to the increased charge-carrier concentration at higher Ni contents. Nevertheless, the incorporation of Ni in the HH phase via 4d site occupation also plays a role, especially at lower Ni concentrations. Based on structural analysis, the solubility limit of Ni interstitials appears to lie between *x* = 0.05 and 0.1, beyond which the FH phase begins to form.

**Figure 6:**
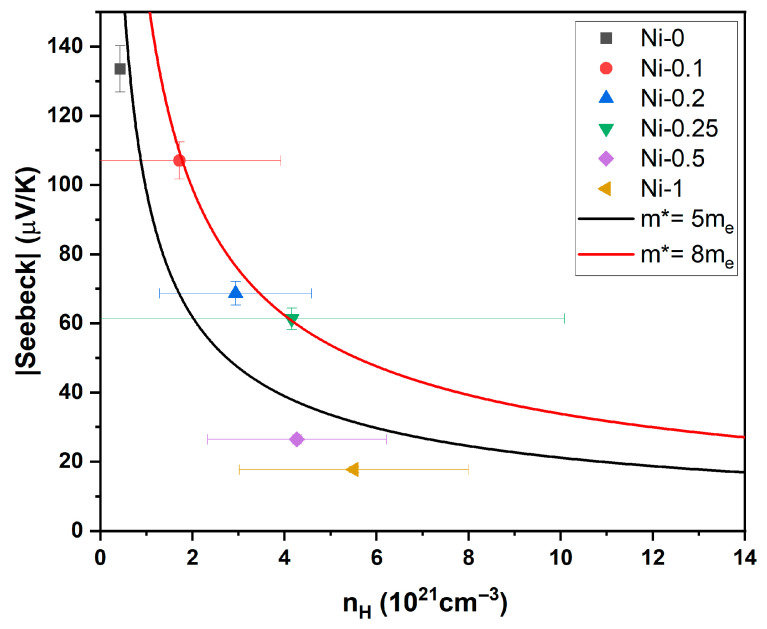
Seebeck coefficient over the Hall carrier concentration for NbCoNi_x_Sn samples at room temperature.

The band structure modification induced by Ni occupation on the 4d site is also reflected in the increase in the charge-carrier effective mass (m*), as shown in Figure 6. The effective mass of sample Ni-0 without any doping showed an effective mass of about 3.7*m_e_*, which is significantly lower than the values reported in the literature for NbCoSn compounds of around 5.4*m_e_* to 6.5*m_e_* [16,24,25]. However, measurement errors from the Hall and Seebeck coefficient measurements are large enough to assume that the presented effective mass is close to the literature values. The samples with high Ni doping content (0.05 < x < 0.5) possess a larger effective mass, which is due to the doping and FH secondary phase [39]. For the samples with x = 0.5 and x = 1, the SPB model may not be valid.

As the charge-carrier concentration increases, a corresponding decrease in mobility is typically expected. This trend is also observed in the present study through the calculated weighted mobility (${µ}_{w}$), as shown in Figure 5b. The weighted mobility was determined using Equation (1) and is preferred over Hall mobility for further analysis, particularly in low-mobility materials where it provides a more reliable representation of carrier transport characteristics [40]. The equation is as follows:

(1) $$\mu_{W}=\frac{3h^{3}\sigma }{8\pi e(2m_{e}k_{B}T{)}^{3/2}}\left[\frac{exp\left(\frac{\left|S\right|}{k_{B}/e}-2\right)}{1+exp\left[-5\left(\frac{\left|S\right|}{k_{B}/e}-1\right)\right]}+\frac{\frac{3}{\pi^{2}}\frac{\left|S\right|}{k_{B}/e}}{1+exp\left[5\left(\frac{\left|S\right|}{k_{B}/e}-1\right)\right]}\right]$$

**Figure 7:**
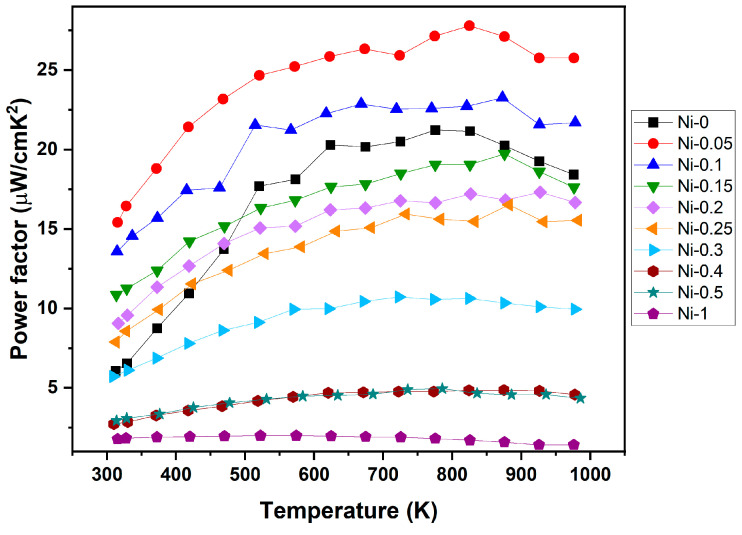
Temperature dependence of the power factor for NbCoNi_x_Sn samples (Ni-*x*).

The weighted mobility initially increases with the addition of Ni but begins to decline as the Ni content is further increased. The initial rise in ${µ}_{w}$ can be attributed to a significant increase in electrical conductivity, while the Seebeck coefficient does not decrease proportionally (Figure 4). This behaviour is consistent with the observed power factor, which is directly influenced by both the electrical conductivity and Seebeck coefficient. As expected from the trend in weighted mobility, the power factor reaches its maximum at low Ni concentrations and then decreases with further Ni additions. The measured power factor values are presented in Figure 7.

The power factors of all samples increase with temperature and reach a plateau at higher temperatures, primarily due to the rising absolute value of the Seebeck coefficient which stabilises at around *T* = 800 K. This behaviour aligns with trends reported in earlier studies on NbCoSn-based compounds [24,25]. A maximum power factor was achieved for the Ni-0.05 sample at 825 K with ~2.77 mW m^−1^ K^−2^. Samples with Ni content up to 0.1 exhibit a higher power factor compared to undoped NbCoSn. However, with further increasing Ni content, power factors decrease almost linearly. This can be attributed to the gradual transition from HH to the FH phase, since the FH NbCoNiSn phase exhibits a much lower Seebeck coefficient and is therefore suboptimal for TE applications. For a higher power factor the Ni content in the range of 0 to 0.1 would have to be finely tuned.

### 3.3. Thermal Transport Properties

**Figure 8:**
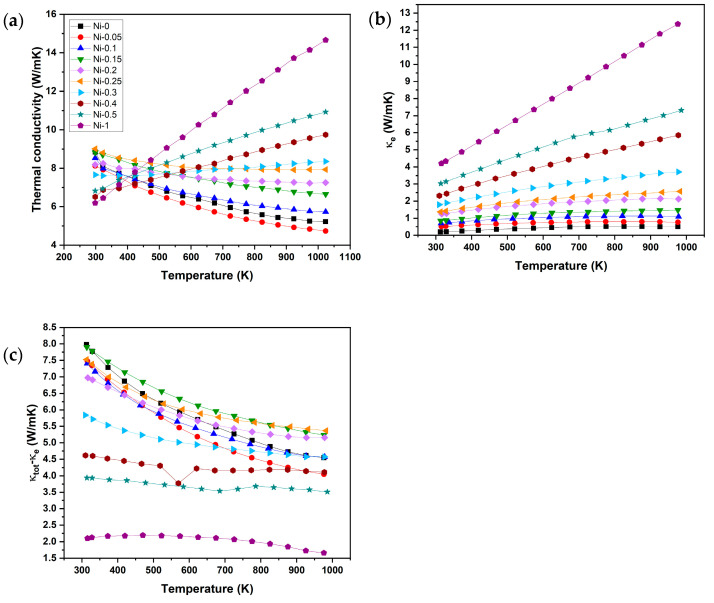
Temperature dependence of (**a**) total thermal conductivity, (**b**) electrical thermal conductivity, and (**c**) bipolar and lattice contribution to the total thermal conductivity of NbCoNi_x_Sn compounds.

The change in total thermal conductivity of NbCoNi_x_Sn is shown in Figure 8a. Compared with undoped NbCoSn (Ni-0), Ni-0.05 possesses the lowest thermal conductivity, and the thermal conductivity of the other Ni-*x´*s steadily increases with higher Ni contents. Moreover, the trend changes, with thermal conductivity increasing with increasing temperature for FH compounds and decreasing with higher temperature for HH compounds. The minimum thermal conductivity achieved was 4.48 W/mK for sample Ni-0.05 at *T* = 1023 K. Results of thermal conductivities for low Ni contents are comparable to the thermal conductivities of previous studies on NbCoSn compounds, ranging between 5 and 10 W m^−1^ K^−1^ [16,24,25,30,31,35,41]. The change in the trend of thermal conductivities is coherent with previously discussed results. Moreover, in comparison to a synthesis procedure without a ball milling step, the total thermal conductivity was reduced while maintaining a high power factor [16].

Figure 8b shows the electrical contribution to the thermal conductivity (*κ_e_ = LσT*, with *L* being the Lorenz number) and the resulting bipolar and lattice thermal contributions. The Lorenz number was obtained according to Equation (2) as follows [42]:

(2) $$L=1.5+exp\left(-\frac{\left|S\right|}{116}\right)$$

With the calculated Lorenz numbers the electrical contribution to the thermal conductivity increases with increased Ni, consistent with the increase in charge-carrier concentration. In contrast, the combination of bipolar and lattice thermal conductivities does not follow the same linear trends as the measurements before. The lattice and bipolar thermal conductivity first decrease with added Ni up to Ni-0.1 and then increase and decrease again with the additional formation of the FH phase. The first decrease is attributed to enhanced phonon scattering caused by Ni-induced point defects in the HH phase, which suppress the lattice thermal conductivity. The subsequent variation is explained by the increasing presence of the FH phase, which alters phonon transport and affects both lattice and bipolar thermal contributions.

### 3.4. Figure of Merit

**Figure 9:**
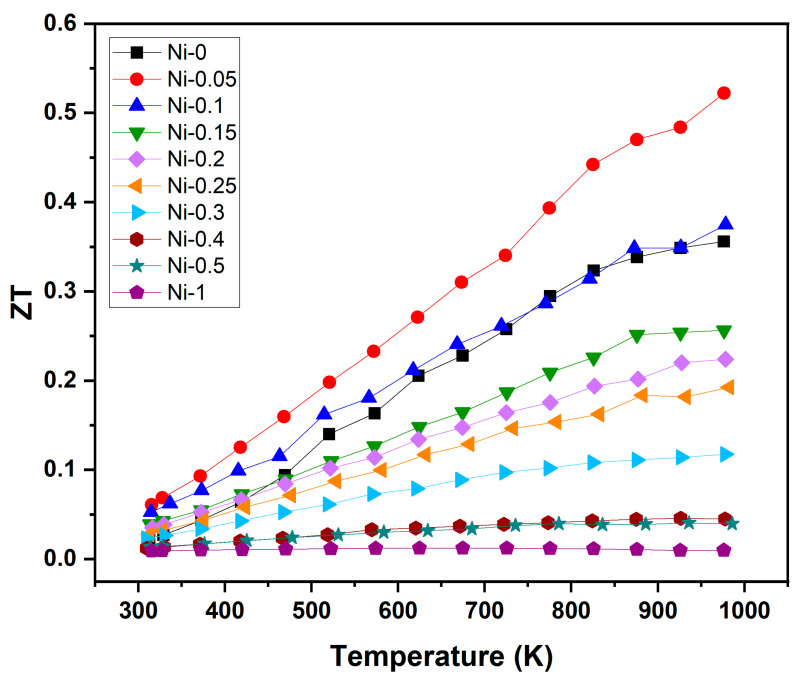
Figure of merit *ZT* of NbCoNi_x_Sn compounds over temperature.

Due to simultaneous improvements of *PF* and reductions in κ, the TE performance (*ZT = S*^2^*σT/κ*) (Figure 9) of the NbCoSn sample was increased for low Ni additions <0.1. A maximum *ZT* value of ~0.52 was achieved at *T* = 975 K for the Ni-0.05 sample, which is an increase of about 48% compared to the pristine NbCoSn sample. Previous studies on NbCoSn compounds indicated a linear increasing trend of *ZT* throughout the measured temperature range [16,24,25,31]. This is only observed for sample Ni-0.05. For all other samples, the TE performance reaches a plateau at temperatures ~850 K without indication of further increases with higher temperatures. Overall, thermoelectric properties could only be enhanced for samples with Ni < 0.1, since an increase in *PF* with a simultaneously small decrease in thermal conductivity was only achieved in these samples. With additional Ni and therefore more of the FH phase, *PF* and thermal conductivity become less suitable for TE application.

## 4. Conclusions

In summary, NbCoSn-based alloys with varying additional Ni content were successfully prepared via arc-melting and high-energy planetary ball milling, followed by SPS and further annealing. The structure and chemical properties of the synthesised samples were systematically characterised and analysed. Ni incorporation was found to increase thermoelectric properties in NbCoSn systems by simultaneously optimising electrical and thermal transport properties. Through controlled Ni addition, occupation of the 4d site was achieved, enabling a gradual transformation from the half-Heusler (HH) to the full-Heusler (FH) phase. This phase evolution was accompanied by corresponding changes in the microstructure and thermoelectric properties. Overall, a maximum power factor of ~2.77 mWm^−1^K^−2^ is obtained at 825 K. Furthermore, the lattice thermal conductivity is decreased due to the enhanced point defect scattering of phonons, leading to a peak *ZT* value of 0.52 at *T* = 975 K in the NbCoNi_0.05_Sn sample.

## Figures and Tables

**Table 1:** Measured densities compared to the theoretical density after SPS of NbCoNi_x_Sn compounds.

Sample	Measured Density [g/cm^3^]	Theoretical Density [g/cm^3^]	Percentage (%)
Ni-0	8.510	8.522	99.86
Ni-0.05	8.537	8.566	99.67
Ni-0.1	8.516	8.610	98.91
Ni-0.15	8.645	8.654	99.90
Ni-0.2	8.668	8.698	99.66
Ni-0.25	8.757	8.742	100.17
Ni-0.3	8.785	8.786	99.99
Ni-0.4	8.828	8.874	99.48
Ni-0.5	8.943	8.962	99.79
Ni-1	9.258	9.401	98.48

**Table 2:** Actual and nominal phase compositions of NbCoNi_x_Sn samples after SPS, measured with EDX.

Nominal Composition	Actual Phase Composition Light Phase	Actual Phase Composition Dark Phase
NbCoSn	Nb_0.89_Co_1.12_Sn	
NbCoNi_0.05_Sn	Nb_0.89_Co_1.12_Ni_0.03_Sn	Nb_0.90_Co_1.12_Ni_0.04_Sn
NbCoNi_0.10_Sn	Nb_0.89_Co_1.12_Ni_0.12_Sn	Nb_0.71_Co_1.12_Ni_0.69_Sn
NbCoNi_0.15_Sn	Nb_0.93_Co_1.04_Ni_0.10_Sn	Nb_0.73_Co_1.35_Ni_0.51_Sn
NbCoNi_0.20_Sn	Nb_0.90_Co_1.10_Ni_0.15_Sn	Nb_0.82_Co_1.67_Ni_0.47_Sn
NbCoNi_0.25_Sn	Nb_0.90_Co_0.97_Ni_0.10_Sn	Nb_0.75_Co_1.30_Ni_0.71_Sn
NbCoNi_0.30_Sn	Nb_0.89_Co_1.04_Ni_0.12_Sn	Nb_0.74_Co_1.60_Ni_0.61_Sn
NbCoNi_0.40_Sn	Nb_0.87_Co_0.98_Ni_0.13_Sn	Nb_0.72_Co_1.37_Ni_0.74_Sn
NbCoNi_0.50_Sn	Nb_0.87_Co_0.99_Ni_0.13_Sn	Nb_0.79_Co_1.35_Ni_0.80_Sn
NbCoNi_1.00_Sn	Nb_0.91_Co_1.10_Ni_1.10_Sn	

## Data Availability

The original contributions presented in the study are included in the article/supplementary material, further inquiries can be directed to the corresponding author.

## Supplementary Materials

The following supporting information can be downloaded at: https://www.mdpi.com/article/10.3390/ma18133189/s1, Figure S1: SEM pictures of fracture surfaces to compare grain sizes of NbCoNi*_x_*Sn compounds. Figure S2: BSE Images (left side) and corresponding Ni elemental EDX mapping (right side).
