# Supplementary material for: Thermoelectric Properties of NbCoNixSn (x = 0–1)
Source: Materials (Basel). 2025 Jul 5;18(13):3189. doi: 10.3390/ma18133189 (PMC12251119; doi:10.3390/ma18133189)
Supplement: Supplementary file 1 [file materials-18-03189-s001.zip › materials-3703480-supplementary.pdf]

Supporting Information

Thermoelectric Properties of $NbCoNi_xSn$ (x = 0–1)

Moritz Thiem, Ruijuan Yan, Anke Weidenkaff and Wenjie Xie *

Materials and Resources, Department of Materials Science, Technical University of Darmstadt, 64287 Darmstadt, Germany

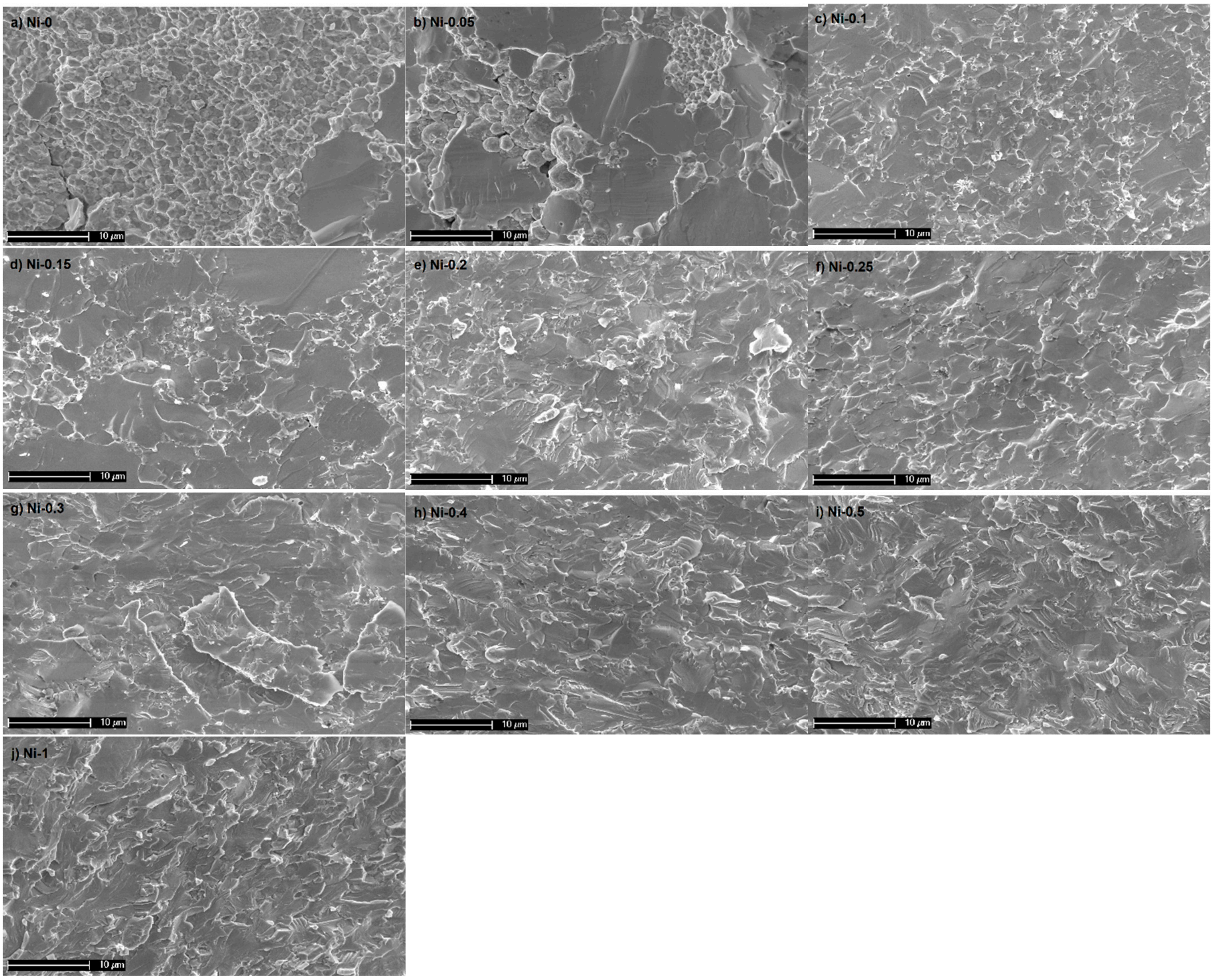

Figure S1. SEM pictures of fracture surfaces to compare grain sizes of $NbCoNi_xSn$ compounds.

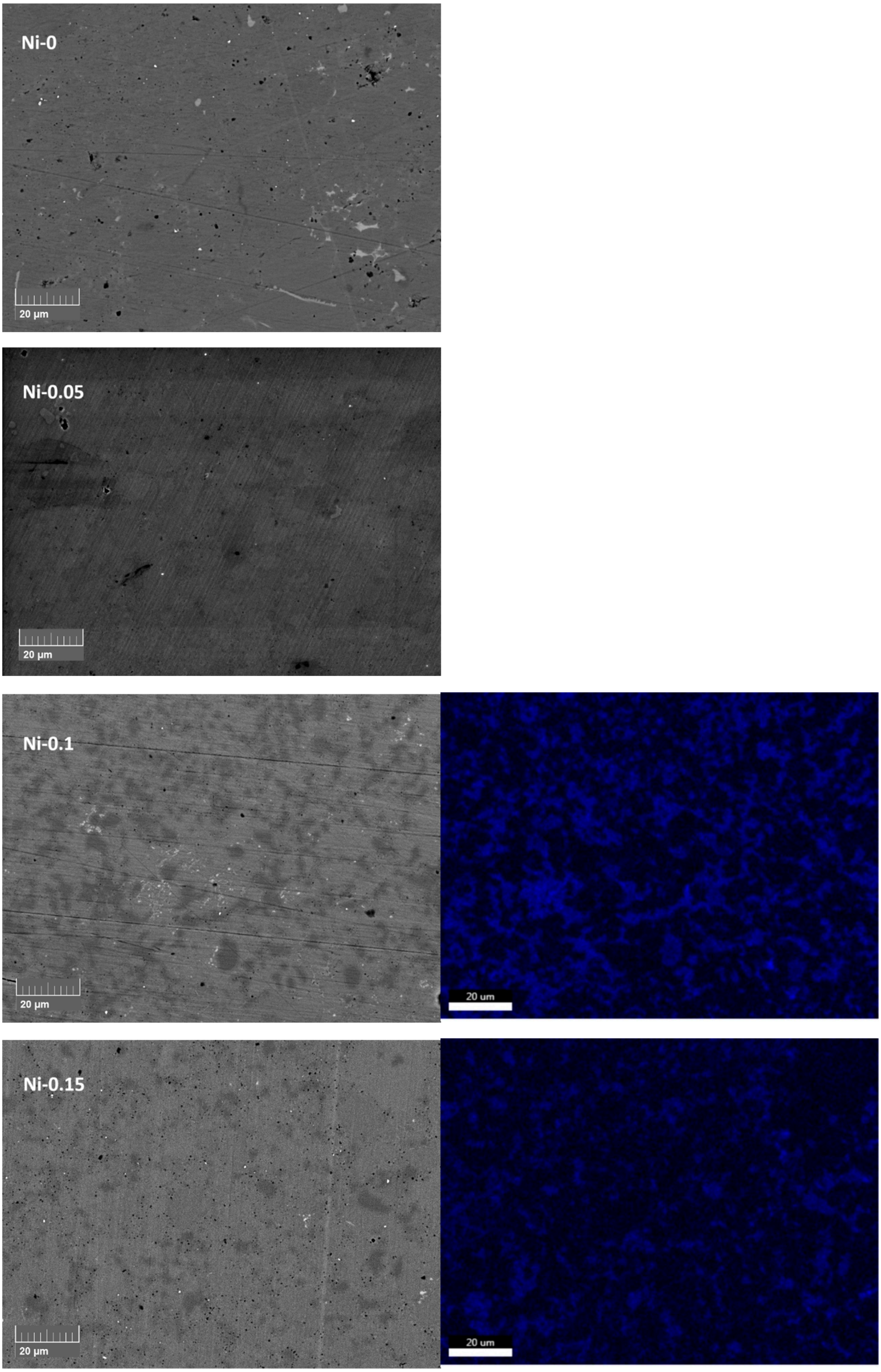
Ni-0
20 µm
Ni-0.05
20 µm
Ni-0.1
20 µm
20 um
Ni-0.15
20 µm
20 um

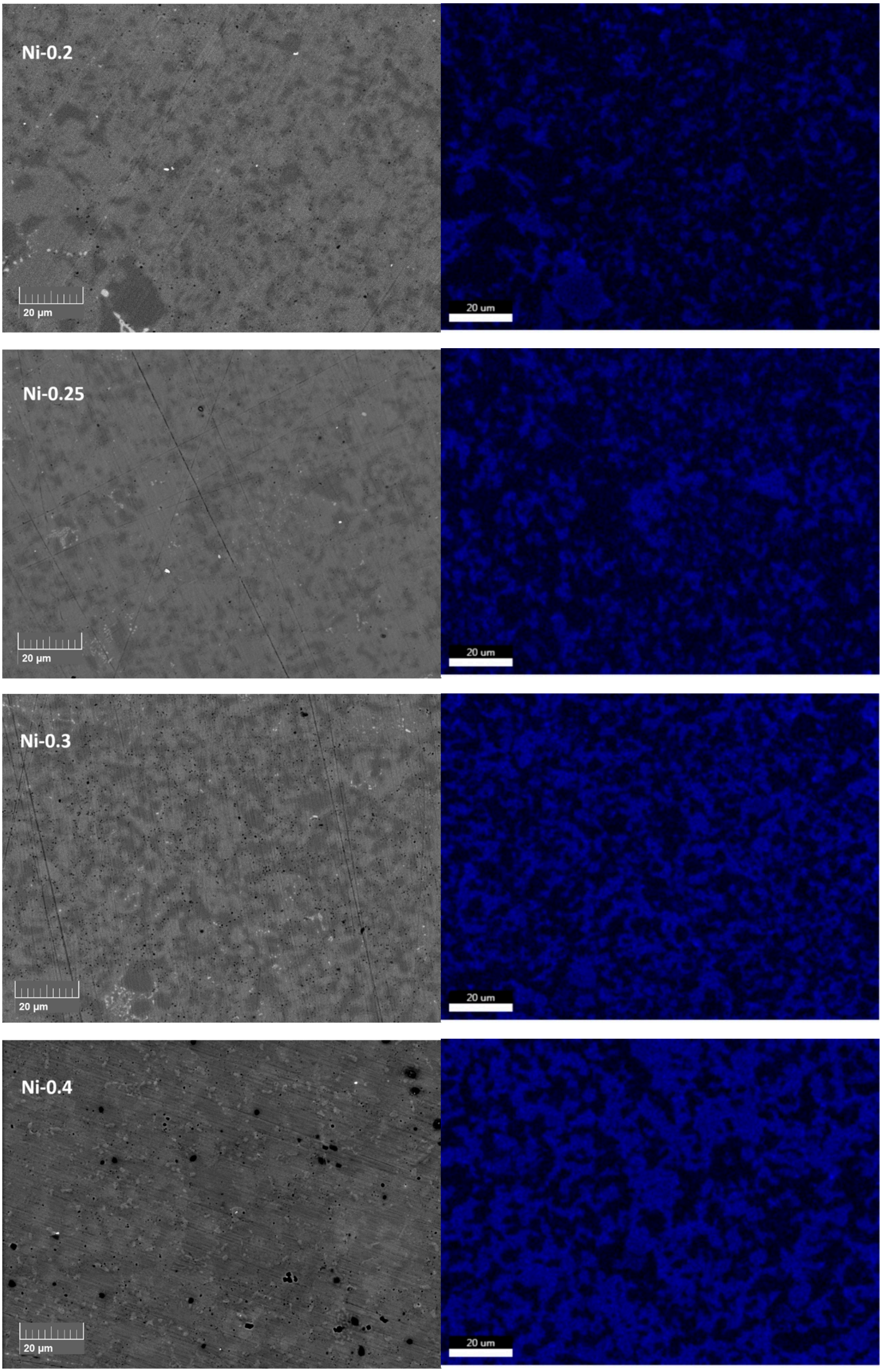
Ni-0.2
20 μm
20 um
Ni-0.25
20 μm
20 um
Ni-0.3
20 μm
20 um
Ni-0.4
20 μm
20 um

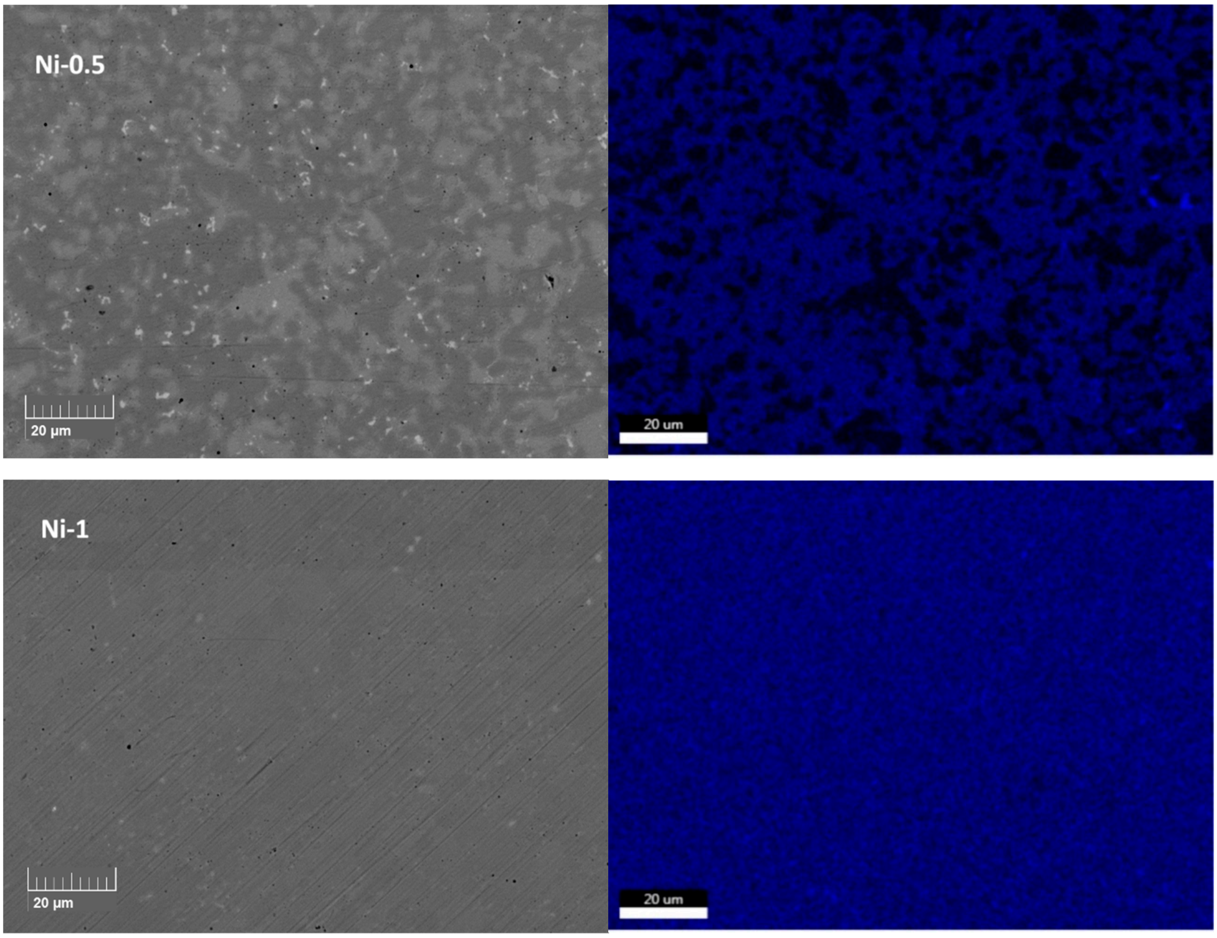

Figure S2. BSE Images (left side) and corresponding Ni elemental EDX mapping (right side)
